# Evaluating the direct effect of vaccination and non-pharmaceutical interventions during the COVID-19 pandemic in Europe

**DOI:** 10.1038/s43856-024-00600-0

**Published:** 2024-09-11

**Authors:** Maxime Fajgenblat, Geert Molenberghs, Johan Verbeeck, Lander Willem, Jonas Crèvecoeur, Christel Faes, Niel Hens, Patrick Deboosere, Geert Verbeke, Thomas Neyens

**Affiliations:** 1 Interuniversity Institute for Biostatistics and Statistical Bioinformatics (I-BioStat), Data Science Institute (DSI), UHasselt, Hasselt, Belgium; 2 Laboratory of Freshwater Ecology, Evolution and Conservation, KU Leuven, Leuven, Belgium; 3 Interuniversity Institute for Biostatistics and Statistical Bioinformatics (I-BioStat), KU Leuven, Leuven, Belgium; 4 Centre for Health Economics Research and Modelling of Infectious Diseases (CHERMID), Vaccine & Infectious Disease Institute (VAXINFECTIO), University of Antwerp, Antwerp, Belgium; 5 Interface Demography (ID), Department of Sociology, Vrije Universiteit Brussel, Brussels, Belgium

**Keywords:** Infectious diseases, Epidemiology

## Abstract

**Background:**

Across Europe, countries have responded to the COVID-19 pandemic with a combination of non-pharmaceutical interventions and vaccination. Evaluating the effectiveness of such interventions is of particular relevance to policy-makers.

**Methods:**

We leverage almost three years of available data across 38 European countries to evaluate the effectiveness of governmental responses in controlling the pandemic. We developed a Bayesian hierarchical model that flexibly relates daily COVID-19 incidence to past levels of vaccination and non-pharmaceutical interventions as summarised in the Stringency Index. Specifically, we use a distributed lag approach to temporally weight past intervention values, a tensor-product smooth to capture non-linearities and interactions between both types of interventions, and a hierarchical approach to parsimoniously address heterogeneity across countries.

**Results:**

We identify a pronounced negative association between daily incidence and the strength of non-pharmaceutical interventions, along with substantial heterogeneity in effectiveness among European countries. Similarly, we observe a strong but more consistent negative association with vaccination levels. Our results show that non-linear interactions shape the effectiveness of interventions, with non-pharmaceutical interventions becoming less effective under high vaccination levels. Finally, our results indicate that the effects of interventions on daily incidence are most pronounced at a lag of 14 days after being in place.

**Conclusions:**

Our Bayesian hierarchical modelling approach reveals clear negative and lagged effects of non-pharmaceutical interventions and vaccination on confirmed COVID-19 cases across European countries.

## Plain language summary

As soon as COVID-19 hit Europe in early 2020, non-pharmaceutical interventions such as movement restrictions and social distancing were employed to contain the pandemic. Towards the end of 2020, vaccination was available and promoted as an additional defence. We analysed almost three years of public COVID-19 data to determine how effective both types of strategies were in containing the pandemic across 38 European countries. We developed a statistical model to relate confirmed cases to how strict non-pharmaceutical interventions were and to vaccination levels. Both non-pharmaceutical interventions and vaccination resulted in decreased confirmed cases, although variation exists among countries. When an intervention is applied, the effect on number of confirmed cases could be seen most about fourteen days after implementation.

## Abstract (web summary)

Fajgenblat et. al utilize almost three years of COVID-19 data to model consequences of interventions across Europe. They find that both non-pharmaceutical interventions and vaccination impact daily case rates, with the strongest effect at a lag of 14 days post-implementation.

## Introduction

When the COVID-19 pandemic hit Europe in the early months of 2020, the only lines of defence took the form of non-pharmaceutical interventions (NPIs), such as mobility restrictions, social distancing, and the closure of schools, economic sectors, and leisure. Very strict and encompassing NPIs are needed for a so-called suppression strategy, whereas they would be relatively lighter for a herd immunity strategy. Most European countries balanced both strategies, following a so-called mitigation strategy, aimed at controlling viral circulation to such an extent that the health care system is protected and burden for the population is kept at a reasonable level^1,2^.

Measures adopted by countries or regions have been diverse and have been changing over time. Depending on the country, NPIs were complemented by vaccination campaigns starting towards the end of 2020 or early 2021. From then onward, there was a gradually increasing fraction of fully vaccinated people^3^. Throughout most of the COVID-19 pandemic in Europe, NPIs have been maintained to a varying extent along with increasing vaccination levels in order to suppress the pandemic^4^.

Quantifying the effectiveness of NPIs and vaccination is of key importance to inform policy-makers. As early as March 2020, evidence for the effectiveness of NPIs was being compiled, emphasising the importance of combining multiple NPIs rather than relying on individual measures^2^. Soon after, Flaxman et al.^5^ demonstrated that NPIs (e.g. national lockdowns) strongly reduced transmission in Europe by developing a Bayesian mechanistic model to link the infection cycle to key health-related and epidemiological outcomes. This seminal study was followed by a multitude of other efforts, varying in spatiotemporal extent, outcome, number and type of interventions, and methodological approach, among other characteristics^6–9^. For instance, a considerable number of studies focused on the effect of facial masking, with substantial evidence for its effectiveness accumulating throughout the first year of the pandemic^9–11^. Other studies addressed compliance to NPIs instead of the effectiveness of NPIs per se and identified considerable variation across space. For instance, Santos et al.^12^ observed very high levels of compliance in Portugal, while lower levels of compliance have been observed in other countries such as in Belgium throughout later stages of the pandemic^13^. Downing et al.^14^ have shown that public compliance varies among NPIs, with the perceived effectiveness being a more important driver compared to one’s fear of contracting COVID-19.

As the availability of data increased throughout the pandemic, large-scale modelling studies gathered increasingly solid evidence on the effectiveness of individual NPIs, with limiting large gatherings, school closings, internal movement restrictions being consistently identified as effective NPIs, along with facial masking^15–17^. Other large-scale studies suggest that combinations of less costly and less intrusive interventions can be equally effective as drastic ones, such as national lockdowns^18^. Sharma et al.^19^ showed that the efficacy of some NPIs dropped after the first wave. For instance, they found school closures to be less effective during resurgent waves, likely due to improved organisational safety measures. The availability of vaccination also impacted the effectiveness of NPIs, with patterns being modulated by or confounded with circulating variants (largely Wild-type in 2020, Alpha, Beta, and Gamma in early 2021 in Europe, Delta in middle and late 2021, etc.^20,21^). For instance, Ge et al.^22^ shows that both NPIs and vaccination jointly act to reduce cases, but that the effectiveness of NPIs decreases with vaccination. They also found the effectiveness of vaccination to be more sensitive to variants compared to the effectiveness of NPIs.

The Stringency Index^4^ has been developed as a convenient summary measure for the package of NPIs adopted by a given country, summarising a country’s approach in those domains that make up the index. These are: school closing (C1); workplace closing (C2); cancelling public events (C3); restrictions on gatherings (C4); closing public transport (C5); stay-at-home requirements (C6); restrictions on internal movement (C7); international travel control (C8); and launching public information campaigns (H1). Each item is scored on an ordinal scale where the number of alternatives is specific to each item. Upon scaling, 0 corresponds to no measures at all along the dimensions present in the index, whereas 100 corresponds to the highest level for all items^4^. As governmental interventions stabilised over the course of 2022 across much of the globe, the Stringency Index is no longer being recorded from 2023 onward^4^. A variety of other initiatives tracking the strength of NPIs have been developed, emphasising different types of NPIs. For instance, the Oxford Covid-19 Government Response Tracker also developed the Comprehensive Health Index, which complements the Stringency Index by also taking into account testing policy, contact tracing, facial covering, vaccination policy and protection of older individuals^4^. The Response Measures Database, developed by the European Centre for Disease Prevention and Control is yet another metric, specifically maintained for 30 European countries^23^. Throughout the remainder of this paper, we will, however, exclusively focus on the Stringency Index, as the Comprehensive Health Index also includes vaccination data, and because the Response Measures Database is only available for a limited number of European countries.

In this paper, we capitalise on almost three years of data on daily incidence, Stringency Index values and vaccination levels to study how the combination of NPIs and vaccination shaped the COVID-19 epidemiological time series of 38 European countries. Rather than proposing a full set of explanatory variables to model the key outcome, our aim is to develop and apply a hierarchical model that comprehensively captures the relationships between these three time-series, flexibly accounting for lagged responses, non-linearities and interactions, as well as for the multi-country setting.

## Methods

### Data selection and preparation

We selected all 38 Pan-European countries with a population size of at least 100,000 inhabitants for which the Stringency Index, the fraction of the total population fully vaccinated, and confirmed cases are available. We retrieved country-level Stringency Index and daily confirmed case data from the Oxford Covid-19 Government Response Tracker project^4^, and country-level data on the fraction of population fully vaccinated (i.e. having received a primary series of COVID-19 vaccines) from OurWorldInData.org^3^ (data retrieval: 17/05/2024).

We smoothed the daily confirmed cases using a centred 7-day rolling average window to address reporting heterogeneity across days of the week. As outcome of interest, we consider the daily confirmed case change *y*_*i *_(*i* = 1, …, *n*), which is calculated as the ratio of the smoothed number of confirmed cases of two subsequent days, subtracted by one. As such, negative values pertain to the percentage decrease in number of cases, while positive values pertain to the percentage increase in number of cases.

As systematic reporting on vaccination in OurWorldInData.org typically only starts from the first non-zero instance onward, we zero-imputed the fraction of population fully vaccinated prior to the first non-zero instance for each country. Furthermore, we used a linear interpolation approach whenever missing data gaps of up to 50 consecutive days occurred in the vaccination time series due to irregular reporting. At the time of data retrieval, data on the fraction of population fully vaccinated was largely missing for the Grand Duchy of Luxembourg and for Switzerland. Instead, we used the fraction of population vaccinated (i.e. at least one dose) for these countries (throughout the entire study period), lagged by a 28-day period to account for the delay between the first and final shot required to achieve a fully vaccinated status. We chose this delay based on guidelines for the interval between doses for several widely used COVID-19 vaccines in these countries, including the Pfizer-BioNTech (3 weeks) and Moderna (4 weeks) vaccines^24,25^. We verified this choice by screening governmental websites and news coverage available for these two countries.

For each country and date, we computed lagged values of the Stringency Index and fraction of population fully vaccinated, in order to determine the temporal delay between interventions and daily confirmed case changes. Specifically, we consider a set of 56 daily lags, ranging from 45 days before the confirmed case change (*t* − 45) to 10 days after the confirmed case change (*t* + 10). The choice for this range is motivated by epidemiological knowledge and has been verified by ensuring that lags beyond this time window feature a negligible importance during preliminary runs. We account for future lags (leads) up to 10 days to assess whether changing interventions are a consequence rather than a cause of confirmed case changes, and to assess whether future interventions might impact confirmed case changes (e.g. through the early adoption of upcoming measures).

Whenever the daily change in confirmed cases or any of the lagged Stringency Index and fraction of the population fully vaccinated values is missing, the data point is omitted from the analysis. In total, *n* = 36, 355 data points across the 38 considered countries, ranging from 27/01/2020 to 28/12/2022, are included in this analysis. A graphical overview of the available data is included in the Supplementary Information (Figs. S1–S2).

### Statistical modelling

We developed a Bayesian hierarchical distributed lag model to relate daily confirmed case changes to past values of the Stringency Index and the fraction of population fully vaccinated in a multi-country setting. The development of this model was driven by two principal aims: (1) to flexibly model the combined effect of both interventions, including non-linearities and interactions, while simultaneously allowing individual countries to deviate from overall patterns, and (2) to enable the model to estimate the varying importance of past and future time lags from the data, rather than requiring strong decisions from the modeller’s side.

Throughout the following, our notation presumes all observed daily confirmed case changes *y*_*i*_ to be arranged in a long vector format, where the observations of all countries are stacked. We use the indexing functions $c\left(i\right)$ and $t\left(i\right)$ to denote the country *c* and day *t* of observation *i* respectively.

We assume each observed daily confirmed case change *y*_*i *_(*i* = 1, …, *n*) to follow a location-scale *t* distribution with *ν* = 3 degrees of freedom:

1 $${y}_{i} \sim {{{\rm{T}}}}_{3}\left({\mu }_{i},{\sigma }_{c(i)}^{2}\right),$$

where *μ*_*i*_ is the linear predictor for observation *i* and *σ*_*c*(*i*)_ is the country-specific scale parameter for the corresponding country *c* of data point *i*. The scale parameters are modelled in a fixed fashion (i.e. a no-pooling scenario). We specifically assume a location-scale *t* distribution as preliminary goodness-of-fit cheques revealed substantial excess kurtosis under a Gaussian model likelihood (Supplementary Information; Fig. S3). We use an identity link function to map the linear predictor to the expected values.

We model the linear predictor *μ*_*i*_ as follows:

2 $${\mu }_{i} \, = \,	 {\beta }_{0}+f\left({\,{{\rm{SI}}}}_{c(i),t(i)}^{* },{{{\rm{Vax}}}\,}_{c(i),t(i)}^{* }\right)\\ 	+{b}_{0,c(i)}+{b}_{{{{\rm{SI}}}},c(i)}\cdot {\,{{\rm{SI}}}\,}_{c(i),t(i)}^{* }+{b}_{{{{\rm{Vax}}}},c(i)}\cdot {\,{{\rm{Vax}}}\,}_{c(i),t(i)}^{* },$$

where *β*_0_ is the overall model intercept, ${\,{{\rm{SI}}}\,}_{c,t}^{* }$ and ${\,{{\rm{Vax}}}\,}_{c,t}^{* }$ are the time-weighted Stringency Index and fraction of population fully vaccinated of country *c* at day *t*, *f* is a two-dimensional smooth function that describes the combined average effect of Stringency Index and the fraction of population fully vaccinated across countries, *b*_0,*c*_ is a random intercept for country *c*, and *b*_SI,*c*_ and *b*_Vax,*c*_ are country-specific random slopes for the effect of Stringency Index and the fraction of population fully vaccinated.

Following a distributed lag modelling approach^26–28^, time-weighted Stringency Indices and fractions of population fully vaccinated are calculated as weighted averages:

3 $${\,{{\rm{SI}}}\,}_{c,t}^{* }= {\sum}_{l=-45}^{10}\left({\gamma }_{l}\cdot {{{\rm{SI}}}}_{c,t+l}\right)\quad \,{{{\rm{and}}}}\,\quad {\,{{\rm{Vax}}}\,}_{c,t}^{* }= {\sum}_{l=-45}^{10}\left({\gamma }_{l}\cdot {{{\rm{Vax}}}}_{c,t+l}\right),$$

where *l* = −45, −44, …, 9, 10 is the lag considered, *γ*_*l*_ is the relative weight of lag *l* and SI_*c*,*t*−*l*_ and Vax_*c*,*t*−*l*_ are the observed Stringency Index and the fraction of population fully vaccinated of country *c* at day *t* − *l*. As indicated, we want the relative lag weights *γ*_*l*_ to be estimated from the data rather than imposing a specific structure from the modeller’s side. We model the raw vector of lag weights ${{{{\boldsymbol{\gamma }}}}}^{{\prime} }={\left({\gamma }_{0}^{{\prime} },{\gamma }_{1}^{{\prime} },\ldots ,{\gamma }_{L}^{{\prime} }\right)}^{\top }$ using a Gaussian process prior (GP^29,30^):

4 $${{{{\boldsymbol{\gamma }}}}}^{{\prime} } \sim {{{\mathcal{GP}}}}\left(0,{k}^{(\gamma )}(\Delta l)\right),$$

where ${k}^{(\gamma )}\left(\Delta l\right)$ is an exponentiated quadratic covariance function that models how similarity in lag weights decays along with an increasing lag distance Δ*l*:

5 $${k}^{(\gamma )}\left(\Delta l\right)={\alpha }^{(\gamma )}\cdot \exp \left(-\frac{1}{2}\cdot {\left(\frac{\Delta l}{{\rho }^{(\gamma )}}\right)}^{2}\right),$$

where *α*^(*γ*)^ is a marginal variance parameter that controls the amplitude in lag weight differences, and *ρ*^(*γ*)^ is a length scale parameter that controls the rate at which the correlation among lags decays along with increasing Δ*l*. The exponentiated quadratic kernel, also known as the radial basis function kernel, is a commonly used kernel in Gaussian process regression and was chosen to ensure smoothness among the highly correlated subsequent lags, as it is infinitely differentiable^31^. To constrain the vector of lag weights ***γ*** to sum to one, the raw lag weights ${{{{\boldsymbol{\gamma }}}}}^{{\prime} }$ are transformed using the *softmax* function:

6 $${\gamma }_{l}=\frac{\exp \left({\gamma }_{l}^{{\prime} }\right)}{{\sum }_{j = 0}^{L}\exp \left({\gamma }_{j}^{{\prime} }\right)}.$$

We assume an identical delay for the effects of the Stringency Index and the fraction of population fully vaccinated. While the model could easily accommodate distinct delays, the delayed effect of vaccination can be expected to be poorly identified due the gradual and monotonous increase in vaccination levels, motivating the assumption of an identical delay.

As both the Stringency Index and the fraction of population fully vaccinated might influence the change in daily confirmed cases *Y* in a non-linear fashion, and as they might interact accordingly, we model their average combined effect across countries through the smooth two-dimensional function *f*. Some regions of the parameter space might be prone to overfitting due to data sparsity, motivating us to model this function using a tensor-product spline surface with a low number (4) of basis functions along each dimension:

7 $$f\left({\,{{\rm{SI}}}}_{c,t}^{* },{{{\rm{Vax}}}\,}_{c,t}^{* }\right)= {\sum}_{i=1}^{4} {\sum}_{j=1}^{4}\left({w}_{i,j}^{(f)}\cdot {B}_{i}^{(f)}\left({\,{{\rm{SI}}}\,}_{c,t}^{* }\right)\cdot {B}_{j}^{(f)}\left({\,{{\rm{Vax}}}\,}_{c,t}^{* }\right)\right),$$

where ${B}_{i}^{(f)}\left(\cdot \right)$ and ${B}_{j}^{(f)}\left(\cdot \right)$ are the *i*’th and *j*’th cubic basis functions with equally spaced knots along each dimension, and ${w}_{i,j}^{(f)}$ is their corresponding weight coefficient.

In addition to the country-specific random intercepts *b*_0,*c*_, we also allow each country to linearly deviate from the overall effect through the country-specific random slopes *b*_SI,*c*_ and *b*_Vax,*c*_. We assume the vector of country-specific effects ${{{{\boldsymbol{b}}}}}_{c}={({b}_{0,c},{b}_{{{{\rm{SI}}}},c},{b}_{{{{\rm{Vax}}}},c})}^{{\top }}$ to follow a multivariate location-scale *t* distribution:

8 $${{{{\boldsymbol{b}}}}}_{c} \sim {{{\rm{MVT}}}}_{\nu }\left({{{\bf{0}}}},{{{\mathbf{\Sigma }}}}\right),$$

with *ν* degrees of freedom (estimated from the data), zero-mean vector ${{{\boldsymbol{0}}}}={\left(0,0,0\right)}^{\top }$ and variance-covariance matrix ${{{\mathbf{\Sigma }}}}={{{\rm{diag}}}}\left({{{\boldsymbol{\tau }}}}\right)\cdot {{{\mathbf{\Omega }}}}\cdot {{{\rm{diag}}}}\left({{{\boldsymbol{\tau }}}}\right)$, where ***τ*** is a vector of scale coefficients and **Ω** is a correlation matrix. By assuming a multivariate location-scale *t* distribution for the random effects, we allow the model to fit deviant country-specific responses while simultaneously preventing these countries from affecting global patterns.

To ascertain that our findings are not affected by confounding caused by temporal dynamics such as the appearance of variants of concern and behavioural changes, we performed a sensitivity analysis by developing a second model that features a country-specific temporal Gaussian process, capturing temporally structured residual patterns. The model structure is identical compared to the main model, except for the additional smooth country-specific functions *g*_*c*_:

9 $${\mu }_{i} = \,	 {\beta }_{0}+f\left({\,{{\rm{SI}}}}_{c(i),t(i)}^{* },{{{\rm{Vax}}}\,}_{c(i),t(i)}^{* }\right) +{b}_{0,c(i)}+{b}_{{{{\rm{SI}}}},c(i)}\cdot {\,{{\rm{SI}}}\,}_{c(i),t(i)}^{* }\\ 	+{b}_{{{{\rm{Vax}}}},c(i)}\cdot {\,{{\rm{Vax}}}\,}_{c(i),t(i)}^{* } +{g}_{c(i)}(t(i)).$$

To ease the computational burden, we use regularised B-splines projected Gaussian processes in a similar fashion as ref. ^32^ instead of exact Gaussian processes, as the latter scale cubically with the number of days. First, we set up 50 basis functions with equally spaced knots along the date range of our study (1067 days in total, i.e. *t* = 1, …, 1067):

10 $${g}_{c}\left(t\right)= {\sum}_{i=1}^{50}\left({w}_{c,i}^{(g)}\cdot {B}_{i}^{(g)}\left(t\right)\right),$$

where ${B}_{i}^{(g)}$ is the *i*’th cubic basis function and ${w}_{c,i}^{(g)}$ is the corresponding weight coefficient for country *c*. We chose 50 basis functions since preliminary runs indicated that this allowed for sufficient temporal resolution. The weight coefficients ${{{{\boldsymbol{w}}}}}_{{{{\boldsymbol{c}}}}}^{{{{\boldsymbol{(g)}}}}}$ for each country are regularised through a Gaussian process prior:

11 $${{{{\boldsymbol{w}}}}}_{{{{\boldsymbol{c}}}}}^{{{{\boldsymbol{(g)}}}}} \sim {{{\mathcal{GP}}}}\left(0,{k}_{c}^{(g)}(\Delta t)\right),$$

where ${k}_{c}^{(g)}\left(\Delta t\right)$ is an exponentiated quadratic covariance function, that models how the covariance among weight coefficients decays along with the number of days Δ*t* that separates their respective knots:

12 $${k}_{c}^{(g)}\left(\Delta t\right)={\alpha }_{c}^{(g)}\cdot \exp \left(-\frac{1}{2}\cdot {\left(\frac{\Delta t}{{\rho }_{c}^{(g)}}\right)}^{2}\right),$$

where ${\alpha }_{c}^{(g)}$ is a country-specific marginal variance parameter that controls the amplitude in temporal patterns, and ${\rho }_{c}^{(g)}$ is a country-specific length scale parameter that controls the rate of temporal turnover.

We assume a weakly informative normal prior for the overall model intercept ${\beta }_{0} \sim {{{\mathcal{N}}}}\left(0,1\right)$, a Lewandowski-Kurowicka-Joe (LKJ) prior for the random effects’ correlation matrix ${{{\mathbf{\Omega }}}} \sim {{{\rm{LKJ}}}}\left(2.0\right)$^33^, a weakly informative half-normal prior for the random effects’ covariance function’s scale parameters ${{{\boldsymbol{\tau }}}} \sim {{{{\mathcal{N}}}}}^{+}\left(0,0.2\right)$, informative inverse-gamma priors ${\rho }^{(\gamma )} \sim {{{\rm{Inv}}}}-{{{\rm{Gamma}}}}\left(5,5\right)$ and ${\rho }_{c}^{(g)} \sim {{{\rm{Inv}}}}-{{{\rm{Gamma}}}}\left(5,5\right)$ to constrain the GPs’ length scales to a sensible range, weakly informative half-normal priors $\sqrt{{\alpha }^{(\gamma )}} \sim {{{{\mathcal{N}}}}}^{+}\left(0,10\right)$ and $\sqrt{{\alpha }^{(g)}} \sim {{{{\mathcal{N}}}}}^{+}\left(0,1\right)$ for the GPs’ marginal standard deviation parameters, and weakly informative half-normal priors for the country-specific residual standard deviation parameters ${\sigma }_{c} \sim {{{{\mathcal{N}}}}}^{+}\left(0,1\right)$. We assume the coefficients of the tensor-product spline basis functions to follow a normal distribution ${{{\boldsymbol{w}}}} \sim {{{\mathcal{N}}}}\left(0,\lambda \right)$, with a weakly informative prior on their scale parameter $\lambda \sim {{{{\mathcal{N}}}}}^{+}\left(0,1\right)$.

### Model implementation

We implemented our Bayesian hierarchical distributed lag model in the probabilistic programming language Stan and performed Markov chain Monte Carlo (MCMC) sampling through the CMDSTANR v.2.31.0 package^34^ in R v.4.2.2 (R Core Team^35^). Stan performs Bayesian inference by means of a dynamic Hamiltonian Monte Carlo (HMC) algorithm, a gradient-based MCMC sampler^36^.

Cubic basis functions for the effect of Stringency Index and fraction of population fully vaccinated level, as well as for the daily basis functions, are computed using the SPLINES package (R Core Team^35^) in R v.4.2.2. The range of the Stringency Index and fraction of population fully vaccinated (expressed as percentage) naturally ranges over [0, 100]. For computational efficiency, we scaled these values to the range [−0.5, 0.5], but we rescaled all output shown to the original scale. For ease of interpretability and compactness, we linearised country-specific responses to the Stringency Index and fraction of population fully vaccinated (i.e. the sum of the smooth function and the effect of the random slopes) through ordinary least squares (OLS) at each posterior iteration. As such, the posterior linear effect of the Stringency Index and fraction of population fully vaccinated can be succinctly visualised for each country. A detailed visual scheme is provided in the Supplementary Information (Fig. S4) to further clarify this procedure.

We ran four MCMC chains of 1000 iterations each, of which the first 500 were discarded as warm-up. As per Stan’s default settings, initial values were uniformly drawn from the [−2, 2]-interval, and were appropriately transformed for constrained parameters. The resulting 2000 posterior samples are summarised using posterior means and 95% equal-tailed credible intervals (bounded by the 2.5% and 97.5% samples from the distribution). We used the TIDYBAYES v.3.0.2 package^37^ to visualise the posterior distributions. We assessed model convergence both visually by means of traceplots and numerically by means of effective sample sizes and the Potential Scale Reduction Factor $\hat{{{{\rm{R}}}}}$, for which all parameters had $\hat{{{{\rm{R}}}}} < 1.1$^38^. We performed posterior predictive cheques, which showed satisfactory goodness of fit. We performed a sensitivity analysis to ensure that the obtained results do not strongly depend on the prior specifications. Visualisations of the traceplots, posterior predictive cheques and the sensitivity analysis are shown in the Supplementary Information (Figs. S5–S8).

The full code for the automated data retrieval pipeline and statistical analysis is available on GitHub through https://github.com/MFajgenblat/covid_stringency_effect and on Zenodo^39^.

### Reporting summary

Further information on research design is available in the Nature Portfolio Reporting Summary linked to this article.

## Results

### Overall effectiveness of interventions

**Fig. 1:**
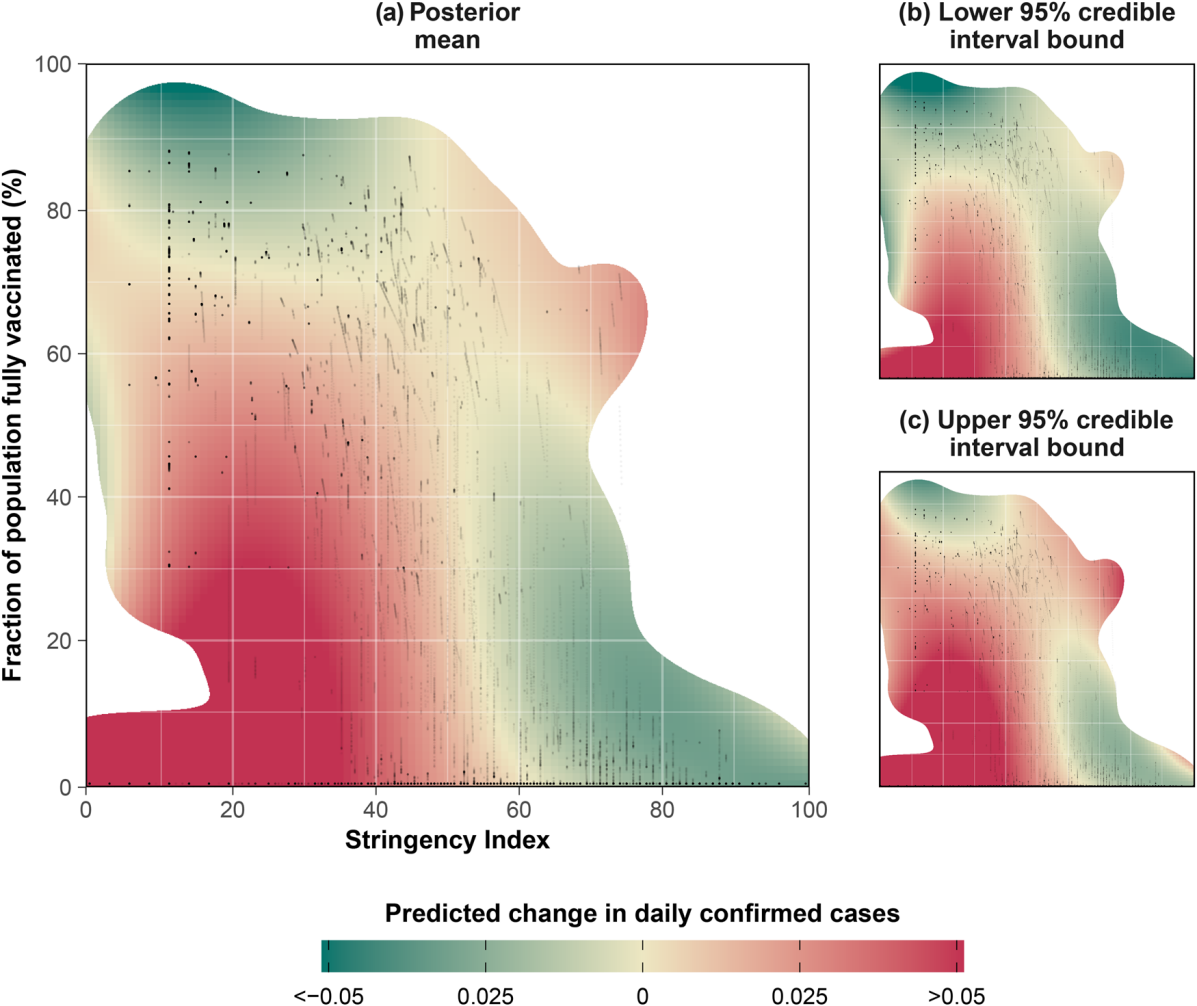
Overall effect of the Stringency Index and vaccination levels. Combined effect of the Stringency Index and the fraction of population fully vaccinated across countries on the daily confirmed case changes, as estimated by the two-dimensional tensor-product spline function *f*, for the average country. Original data points are shown as black dots. Zones with no or poor data availability (defined as the lower tercile region of a bivariate normal kernel density smooth of the original data points, with bandwidth 20) have been whited out as these zones pertain to mere extrapolation, preventing meaningful inference. **a** displays posterior mean predictions, (**b**) displays the lower bounds of 95% credible intervals, and (**c**) displays the upper bounds of 95% credible intervals. Green zones pertain to combinations of the Stringency Index and the fraction of population fully vaccinated that are expected to confer an absolute reduction in daily confirmed cases, while red zones are expected to confer an absolute increase. Yellow zones correspond to a zero change in confirmed cases.

We estimated the combined effect of the Stringency Index and the fraction of population fully vaccinated on the change in daily confirmed cases across 38 European countries using almost three years of data. Our model captures the combined average effect of both intervention measures across countries by means of a two-dimensional response surface, shown in Fig. 1.

Qualitatively, a low Stringency Index combined with a low fraction of population fully vaccinated is linked to very strong increases in daily confirmed cases (change in daily confirmed cases > 0) (Fig. 1; lower left corner). Higher values of the Stringency Index or the fraction of population fully vaccinated are expected to lower the daily confirmed case changes (Fig. 1; right and upper half). Beyond a certain point, high values for the Stringency Index or for the fraction of population fully vaccinated are expected to confer a reduction in the daily confirmed cases (change in daily confirmed cases < 0). High values of the Stringency Index combined with a high fraction of population fully vaccinated, however, feature a positive change in daily confirmed cases (Fig. 1; upper right corner). Importantly, the absence of data in large zones of the two-dimensional stringency-vaccination space (especially across countries) leads to a high level of uncertainty and prevents meaningful inference in these zones of the response surface (Fig. 1).

### Inter-country heterogeneity in effectiveness

**Fig. 2:**
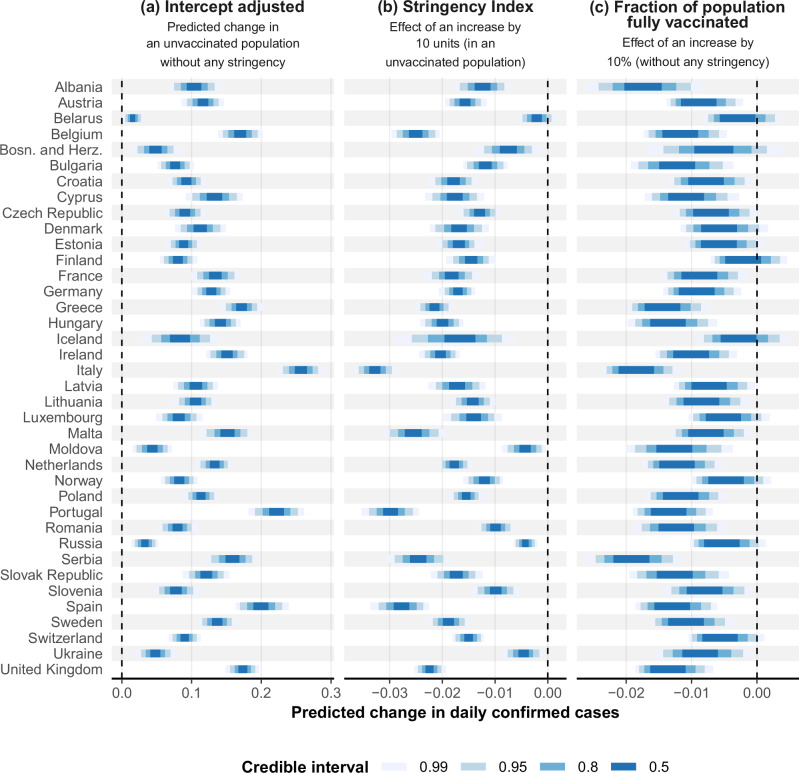
Inter-country heterogeneity in the effect of the Stringency Index and vaccination levels. Country-specific estimates for the intercept (**a**), the effect of the Stringency Index (**b**) and the fraction of population fully vaccinated (**c**), represented by 50, 80, 95 and 99% credible intervals. For the sake of interpretation, the shown intercept values are adjusted to represent the predicted change in daily confirmed cases when the Stringency Index and the fraction of population fully vaccinated equal 0. The country-specific effects of the Stringency Index and the fraction of population fully vaccinated are derived from the smooth two-dimensional function *f* (describing common patterns across countries) and the country-specific random slopes *b*_*s*,*c*(*i*)_ and *b*_*v*,*c*(*i*)_, and have been linearised using ordinary least squares (at each posterior iteration) for the ease of interpretability and compactness. Their value represents the predicted change in daily confirmed cases upon a ten-unit change in the Stringency Index or a 10 percentage point change in the fraction of population fully vaccinated, conditionally on a 0 value for the other variable. A schematic overview of the procedure followed to derived the linear slopes is provided in the Supplementary Information (Fig. S4).

We observe important differences in the effects of the Stringency Index among countries (Fig. 2). All countries show a clear negative (>99% posterior probability) but highly variable effect, with the exception of Belarus, for which the evidence of a negative effect is lower (92.8% posterior probability). This variability is reflected by the large scale coefficient *τ*_2_ (posterior mean 0.070, 95% CrI [0.052, 0.091]) for the country-specific random slopes of the Stringency Index, as well as by the low estimated degrees of freedom *ν* for the multivariate location-scale *t* distribution used to model the country-specific effects (posterior mean 19.352, 95% CrI [4.569, 51.787]). The three countries with the strongest effect of the Stringency Index are Italy, Portugal, and Spain. In these countries, raising the Stringency Index by ten units unit confers a posterior mean reduction in the change in daily confirmed cases by 0.033, 0.030 and 0.028, respectively. Though seemingly small, these estimated effects can be highly effective to control an epidemic as the Stringency Index can be raised by more units, and as their effect is multiplicative over the course of days during which a given level of stringency is held.

Belarus, Russia, Moldova and Ukraine are the countries with the weakest negative effects. In these countries, raising the Stringency Index by ten units confers a posterior mean reduction in the change in daily confirmed cases by only 0.002, 0.004, 0.004 and 0.005, respectively.

Similarly to the Stringency Index, the fraction of population fully vaccinated shows a clear negative effect across most countries. Though inter-country variation is also present, it is noticeably lower compared to the variation present in the country-specific responses to the Stringency Index. This is reflected by the random effect’s scale coefficient *τ*_3_ (posterior mean 0.042, 95% CrI [0.031, 0.055]), with > 99.9% posterior probability of this coefficient being lower than the one that captures inter-country variation in response to the Stringency Index. The countries Iceland, Finland, and Belarus display a lower statistical support for a negative effect compared to the other countries.

Our findings are robust against altered modelling decisions, as demonstrated by multiple sensitivity analyses. First, prior sensitivity analyses demonstrate that alternative prior specifications yield almost identical posterior distributions of model parameters (Supplementary Information; Fig. S7). Second, the omission of outlying countries such as Belarus does not meaningfully impact findings for the other countries (Supplementary Information; Figs. S8–9). Third, an alternative model specification in which temporally structured residual variation is modelled through country-specific Gaussian processes, yields quantitatively different but qualitatively consistent patterns (Supplementary Information; Fig. S10), despite identifying strong temporal patterns for some countries (Supplementary Information; Fig. S11).

### Delayed effectiveness of interventions

**Fig. 3:**
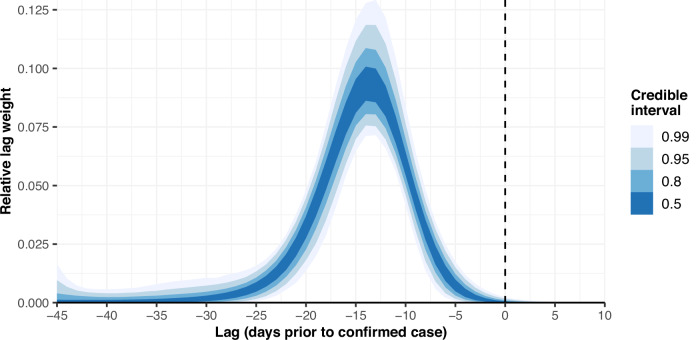
Delay in the effectiveness of interventions. Estimated delay in the effect of the Stringency Index and the effect of the fraction of the population fully vaccinated on the change in daily confirmed cases, modelled as a Gaussian process and visualised by means of 50, 80, 95 and 99% credible intervals. For each considered lag *l*, the curve represents the estimated relative weight *γ*_*l*_.

The estimated delay in the effect of the Stringency Index and the fraction of the population fully vaccinated is shown in Fig. 3. The posterior median lag with the highest relative weight equals 14 days prior to the confirmed case (95% CrI [12, 15]), which corresponds to the amount of time required for interventions to impact the change in daily confirmed cases most. 95% of the cumulative lag weight is reached by day 24 (95% CrI [20, 33]), indicating that interventions do not meaningfully impact the change in daily confirmed cases afterwards. A posterior median fraction of 0.03% (95% CrI [0.00, 0.34]) of the lag weight is allocated to positive lags, indicating that the confirmed case changes are almost exclusively influenced by past interventions, rather than by upcoming interventions. When interpreting the delay between interventions and the daily confirmed case changes, it is important to note that our estimates also include an administrative delay, as the official registration of a confirmed case is typically lagged compared to the true onset of infection. However, our delay estimates do not feature any additional lag due to data preparation, as we used a centred 7-day rolling average window to smooth out intra-week reporting heterogeneity.

## Discussion

Our analysis reveals a strong link between the change in daily confirmed cases and the Stringency Index and the fraction of population fully vaccinated across Europe. Furthermore, the estimated delay function shows that past values of the Stringency Index are the most important in influencing present cases, hinting at the causal direction of the relationship between governmental interventions and the change in daily confirmed cases.

The effect of the Stringency Index varies strongly across countries. Strong effects are beneficial as a lower Stringency Index is sufficient to keep the epidemic under control. Variation in effect strength can be due to, among others, ineffective measures, slow implementation, too frequent changes, civil inertia, civil disobedience and pandemic fatigue^40,41^. The effectiveness of NPIs as measured by the Stringency Index can be strongly influenced by the level of population compliance. For instance, a study on compliance in Portugal indicates high adherence to NPIs, which likely enhances their effectiveness^12^. In contrast, lower compliance in other countries could diminish the impact of similarly stringent measures, thus affecting the overall relationship between the Stringency Index and changes in daily confirmed cases. Low apparent effectiveness might also be due to the effectiveness of voluntary behavioural changes^42^.

The countries Portugal, Italy, and Spain display the highest effectiveness with respect to the Stringency Index, meaning that relatively little interventions are sufficient to prevent confirmed cases from rising. Several factors likely contribute to this observation. Italy was one of the first European countries to be severely impacted by COVID-19 and implemented stringent measures early on, which remained in place over time. Similarly, Spain experienced significant outbreaks, including during the summer of 2021 due to tourism, but Spanish residents continued to adhere to precautionary measures. Both countries maintained relatively stable and consistent interventions, unlike some other European nations, such as the Czech Republic, where measures fluctuated more. Portugal, being geographically isolated in the southwestern corner of Europe, experienced less transit traffic compared to countries such as Italy or Spain. This isolation, combined with prolonged and robust governmental measures, resulted in high public trust and a notably high vaccination rate. Portugal also implemented unique policies, such as differentiated measures for weekdays and weekends, which allowed essential activities like work and school to continue while reducing non-essential movement. For instance, inter-regional travel was permitted during the week but restricted on weekends, effectively limiting occasional interactions that would have otherwise substantially contributed to transmission. These strategic and consistent approaches likely played a crucial role in the higher effectiveness of NPIs observed in these countries.

Vaccination helps to bring down the required non-pharmaceutical interventions (NPIs; which are expressed in the Stringency Index) to manage the epidemic^22^. Vaccination is especially effective when a high percentage of the population has been vaccinated (herd immunity^43^). Hence, aiming for the higher vaccination levels can be beneficial, even (especially) if the vaccination levels are already high. The effect strength of vaccination also varies across countries. In line with Ge et al.^22^, our findings indicate that the effectiveness of NPIs decreases with increasing vaccination levels. Additionally, we observe that the combination of high stringency and high vaccination levels is linked to increasing changes in confirmed cases. This outcome can be attributed to several countries that experienced severe outbreaks from September 2021 to March 2022 despite high vaccination rates, including Austria, Cyprus, Italy, The Netherlands, Norway, and Switzerland. During this period, attempts to suppress these outbreaks through NPIs had limited success. This is likely due to a combination of factors, including the emergence of variants of concern, notably the highly transmissible and immune-escaping Omicron variant^20,44,45^, and pandemic fatigue, which resulted in reduced compliance with NPIs^21,40^. Overall, mechanisms such as the appearance of variants of concern and the reduced compliance with NPIs can be expected to confound the effectiveness of vaccination levels regardless of the Stringency Index, leading to apparent reduced effectiveness. This mechanism might be responsible for the weak relationship between vaccination and confirmed case changes in Norway and Iceland as identified in our study, while other more targeted studies did demonstrate a strongly beneficial effect of vaccination on public health in these countries^46^.

Variation in the effectiveness of vaccination can also arise from differences in vaccination policies^47^. In our analysis, countries featuring successful prioritisation strategies are expected to display a higher estimated effectiveness of vaccination compared to other countries. Time-varying differences in vaccination policies among countries, however, can be expected to yield patterns of temporally changing effectiveness, which are left unexplained by the model as countries are only allowed to deviate in a linear fashion from overall patterns through random slopes.

Throughout the analysis, Belarus was identified as a strongly deviant country with respect to the effects of interventions. This country displays weak links between the Stringency Index and the fraction of population fully vaccinated on the one hand, and the confirmed case changes on the other hand. Using reported COVID-19 cases is challenging as these have largely been underestimated in many countries^48,49^. Given the number of countries and the long observation period involved, we estimate that the overall effect is relatively well captured, but spatial and temporal patterns in under-reporting will likely contribute to the large variation in effect between countries and might partly explain the deviant result for Belarus. For instance, note that the country’s number of reported cases remains very flat and almost never exceeds 200 per million before reaching a peak in March 2022. Additionally, extreme differences exist between the reportedly low COVID-19 mortality and excess mortality reaching 70% at the end of 2020^50^. On 31 March 2021 (last available data), Belarus reports a total of 235 COVID-19 deaths per million inhabitants, compared to a cumulative excess mortality of 3.274 deaths per million for the same period^50^. Accordingly, under-reporting and lack of accuracy in reporting daily cases is very probable. The model we developed is, however, robust against such issues due to the use of a location-scale *t* distribution. This approach effectively reduced the impact of outlying countries, as demonstrated by the unaffected results when excluding Belarus from the analysis (Supplementary Information; Figs. S8–S9), and highlights the merits of heavy-tailed distributions when analysing complex multi-country.

As opposed to the weak effects of the Stringency Index and the fraction of population fully vaccinated, Belarus features the lowest intercept of all considered countries and, hence, shows the smallest positive change in daily confirmed cases in the complete absence of interventions. Even if this pattern did not solely arise from poor data quality, the ability of a country to (better) keep the epidemic situation under control without any intervention (through, for example, the buildup of natural immunity) is not necessarily beneficial as it might have detrimental consequences beyond the registered confirmed cases. Indeed, several lines of evidence suggest that Belarus faced a strongly increased excess mortality compared to other European countries^51^.

The Stringency Index and the fraction of population fully vaccinated influence the daily confirmed cases most with a lag of approximately 14 days, and their effect is estimated to be negligible beyond ~24 days prior to confirmed cases (<5% of cumulative lag weight). Policymakers reacting to peaking confirmed cases by imposing additional NPIs might induce an apparent but non-causal relationship between confirmed case changes and the Stringency Index. While single-lag analyses would fail to rule out this mechanism, our distributed lag approach enabled us to show that this mechanism is unlikely, as the amount of weight assigned to future lags is negligible. Nevertheless, we found that past lags as small as 5 days still hold non-negligible importance. Given that the combined duration of the incubation period, the time from symptom onset to diagnosis, and the reporting delay typically amount to considerably longer delays, this suggests that interventions might already impact transmission before their official implementation. This might be due to early adoption following the communication of upcoming interventions.

Despite the clear findings, it is important to acknowledge a number of limitations of this study. For instance, the use of the Stringency Index precludes us from determining the effectiveness of individual NPIs or the interactions among them. Examining the heterogeneity in the effectiveness of individual NPIs across different countries would be particularly valuable, as it could provide insights into the optimal implementation of various types of NPIs. Moreover, the change in daily confirmed cases is affected by a multitude of other phenomena, including endogenous epidemiological processes, meteorological factors, emerging variants of concern, temporal variation in testing policy and behaviour, waning immunity, stringency of governmental measures not properly captured by the Stringency Index (e.g. face mask covering mandates), some of which might have a confounding effect or might invoke complex feedback loops^20,28,48,49,52–56^. Though disentangling the relative importance of these drivers is beyond the scope of this paper, an alternative model that accounts for temporal dynamics yields similar results, and our model could easily be extended to include additional predictors. The use of confirmed cases might also blur epidemic patterns, for instance when testing capacity does not scale appropriately during strong epidemic growth, potentially affecting model estimates. Finally, the data are relatively sparse as there are, e.g., only a few instances where a high Stringency Index and a high vaccination rate co-occur. This makes it difficult to model non-linear effects in more detail across the entire intervention space.

The Stringency Index effectively communicates a summary of the intensity and comprehensiveness of government responses to COVID-19 over time and across countries. By aggregating multiple dimensions of NPIs into a single metric, the index facilitates comparisons of policy strictness between countries over time. However, it does not account for the compliance with these measures, nor does it capture the nuances of how these policies are implemented or enforced. Moreover, the Stringency Index only summarises a subset of the broad set of possible interventions, and does not, for instance, feature facial masking. In conclusion, the Stringency Index is an aggregate measure with many disadvantages, but this study shows that there is a strong relationship between a country’s NPIs (translated in the Stringency Index) and its ability to control the epidemic.

## Supplementary information

Supplementary Information

Reporting Summary

## Data Availability

Source data used in this work can be retrieved online through the Oxford Covid-19 Government Response Tracker’s GitHub repository (Stringency Index and daily confirmed cases^4^) and from OurWorldInData.org’s GitHub repository (fraction of population fully vaccinated^3^).

## Code availability

Fully documented code for automated source data retrieval and for the statistical analysis is available on GitHub through https://github.com/mfajgenblat/covid_stringency_effect and on Zenodo^39^.
