# Supplementary material for: Evaluating the direct effect of vaccination and non-pharmaceutical interventions during the COVID-19 pandemic in Europe
Source: Commun Med (Lond). 2024 Sep 11;4:178. doi: 10.1038/s43856-024-00600-0 (PMC11391057; doi:10.1038/s43856-024-00600-0)
Supplement: Supplementary file 1 — Supplementary Information [file 43856_2024_600_MOESM1_ESM.pdf]

Evaluating the direct effect of vaccination and non-pharmaceutical interventions during the COVID-19 pandemic in Europe.

SUPPLEMENTARY INFORMATION

Maxime Fajgenblat[1,2*], Geert Molenberghs[1,3], Johan Verbeeck[1], Lander Willem[4], Jonas Crèvecoeur[1,3], Christel Faes[1], Niel Hens[1,4], Patrick Deboosere[5], Geert Verbeke[1,3], Thomas Neyens[1,3]

[1*]Interuniversity Institute for Biostatistics and statistical Bioinformatics (I-BioStat), Data Science Institute (DSI), UHasselt, Hasselt, Belgium.
[2]Laboratory of Freshwater Ecology, Evolution and Conservation, KU Leuven, Leuven, Belgium.
[3]Interuniversity Institute for Biostatistics and statistical Bioinformatics (I-BioStat), KU Leuven, Leuven, Belgium.
[4]Centre for Health Economics Research and Modelling of Infectious Diseases (CHERMID), Vaccine & Infectious Disease Institute (VAXINFECTIO), University of Antwerp, Antwerp, Belgium.
[5]Interface Demography (ID), Department of Sociology, Vrije Universiteit Brussel, Brussels, Belgium.

*Corresponding author(s). E-mail(s): maxime.fajgenblat@gmail.com;

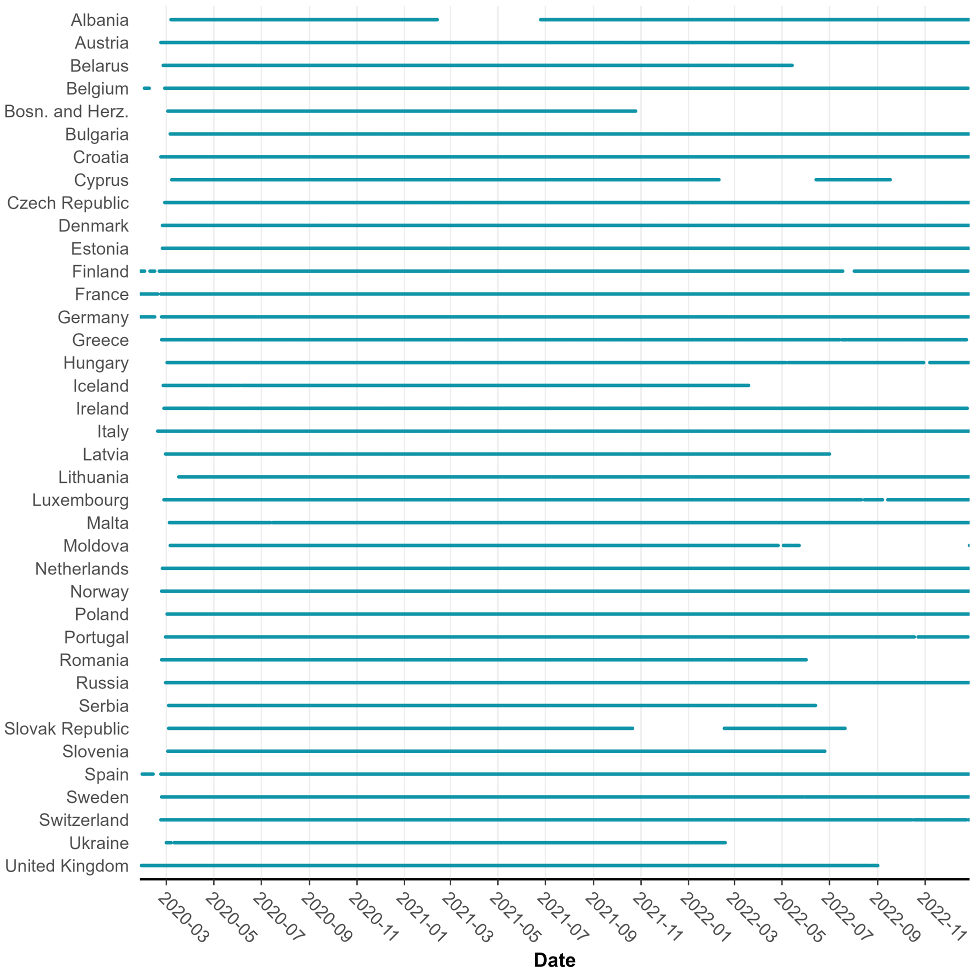

Fig. S1 Overview of the available data for all considered countries. The availability of data for a particular day and country is shown by means of a blue dot.

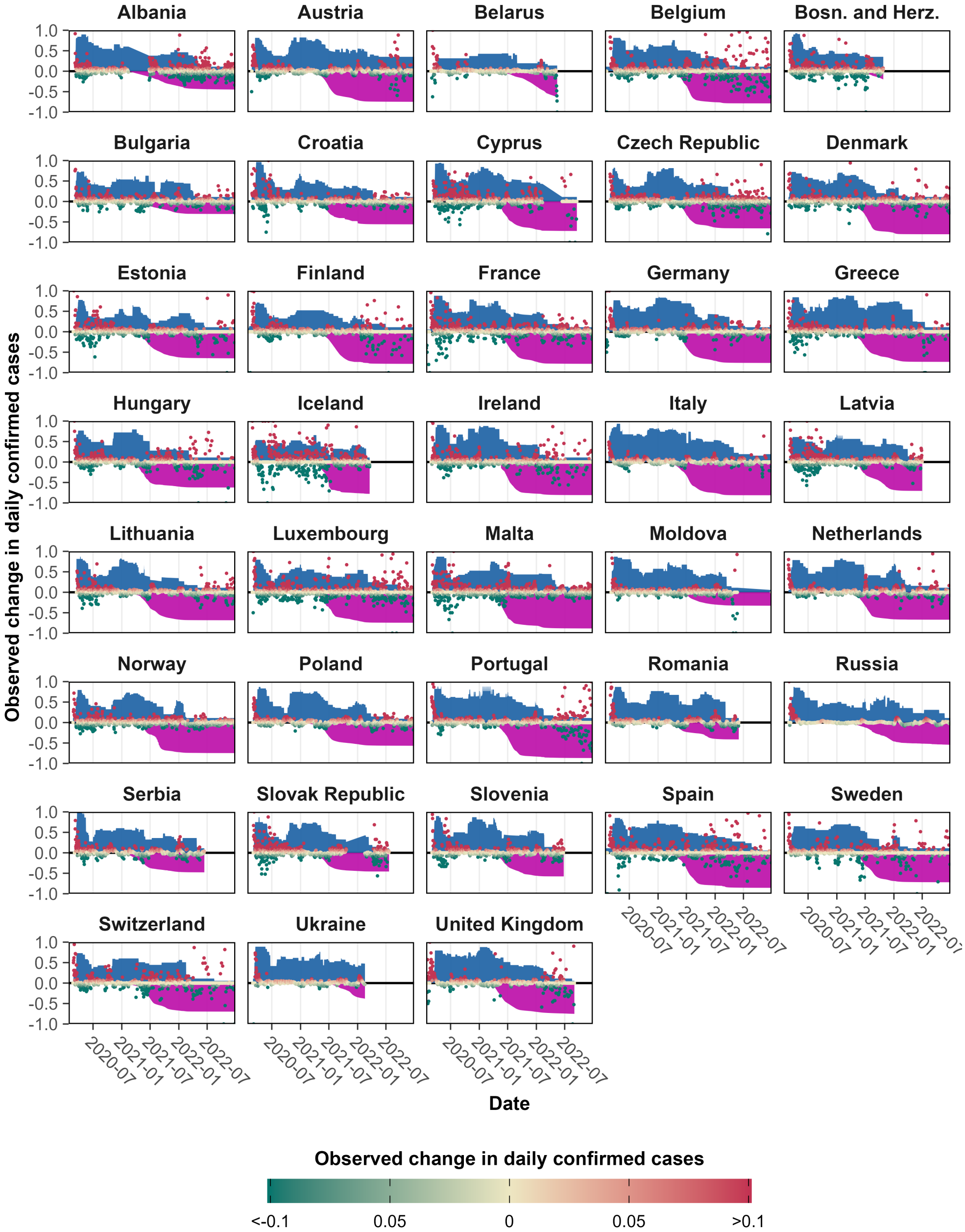

Fig. S2 Observed changes in daily confirmed cases throughout the study period across all countries (dots). In addition to the vertical positioning, a colour scale is used to indicate the direction and magnitude of the change. The blue shaded zones depict the Stringency Index for each country and time point, scaled between 0 (Stringency Index = 0) and 1 (Stringency Index = 100). The pink shaded zones depict the fraction of population fully vaccinated for each country and time point, scaled between 0 (0% fraction of population fully vaccinated) and -1 (100% fraction of population fully vaccinated).

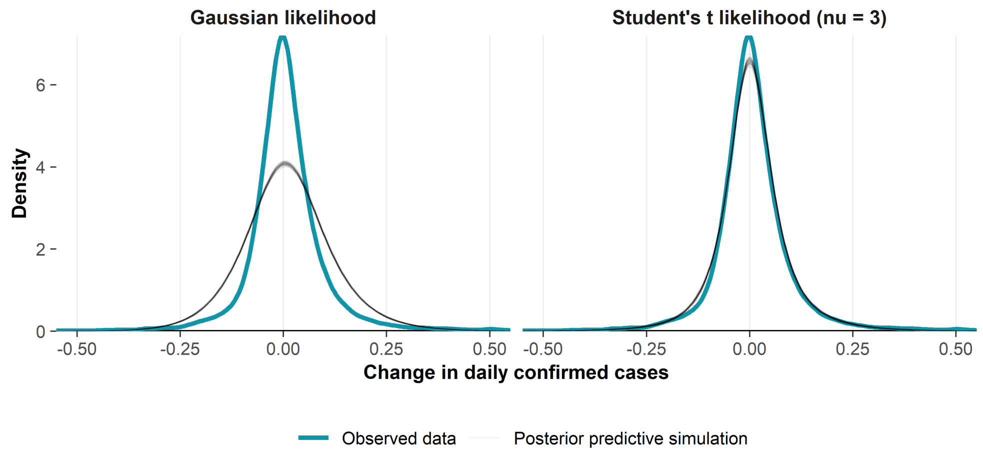

Fig. S3 Posterior predictive checks. The solid blue line shows the observed density of changes in daily confirmed cases, while the transparent black lines show the posterior predictive simulations for the different MCMC draws, thinned to every tenth iteration. The left panel shows results for a model version featuring a Gaussian likelihood and reveals substantial kurtosis. The right panel shows results for the main model (considered in the paper) featuring a Student's t model likelihood with 3 degrees of freedom, yielding a strongly improved goodness-of-fit.

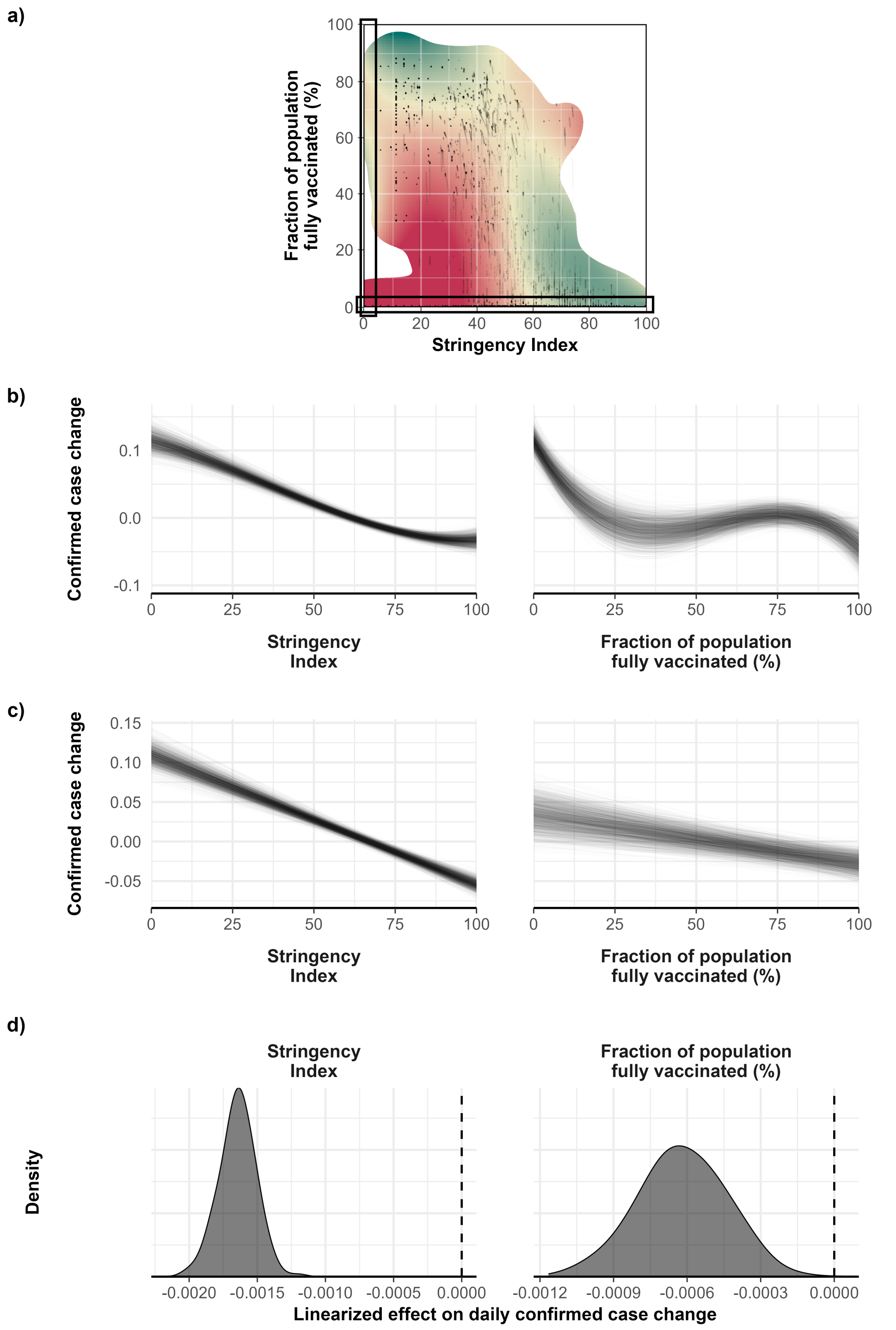

Fig. S4 Schematic overview of the procedure to derive the linear slopes depicted in Figure 2. (a) The country-specific reaction surfaces form the starting point of the procedure. The black-bordered rectangles indicate the regions for which the linearisation procedure will be used. (b) From (a), the posterior functional relationships between the Stringency Index and the confirmed case changes for a zero fraction of population fully vaccinated on the one hand, and between the fraction of population fully vaccinated and the confirmed case changes for a zero fraction of population fully vaccinated. Each individual posterior draw is represented by a transparent black line. (c) Each of the functional curves in (c) is linearised through ordinary least squares, yielding a line for each posterior draw. (d) From the posterior linearised relationships in (c), the posterior distribution of the slopes can be computed. These posterior slopes are further summarised through 50, 80, 95 and 99% credible intervals and depicted in Figure 2.

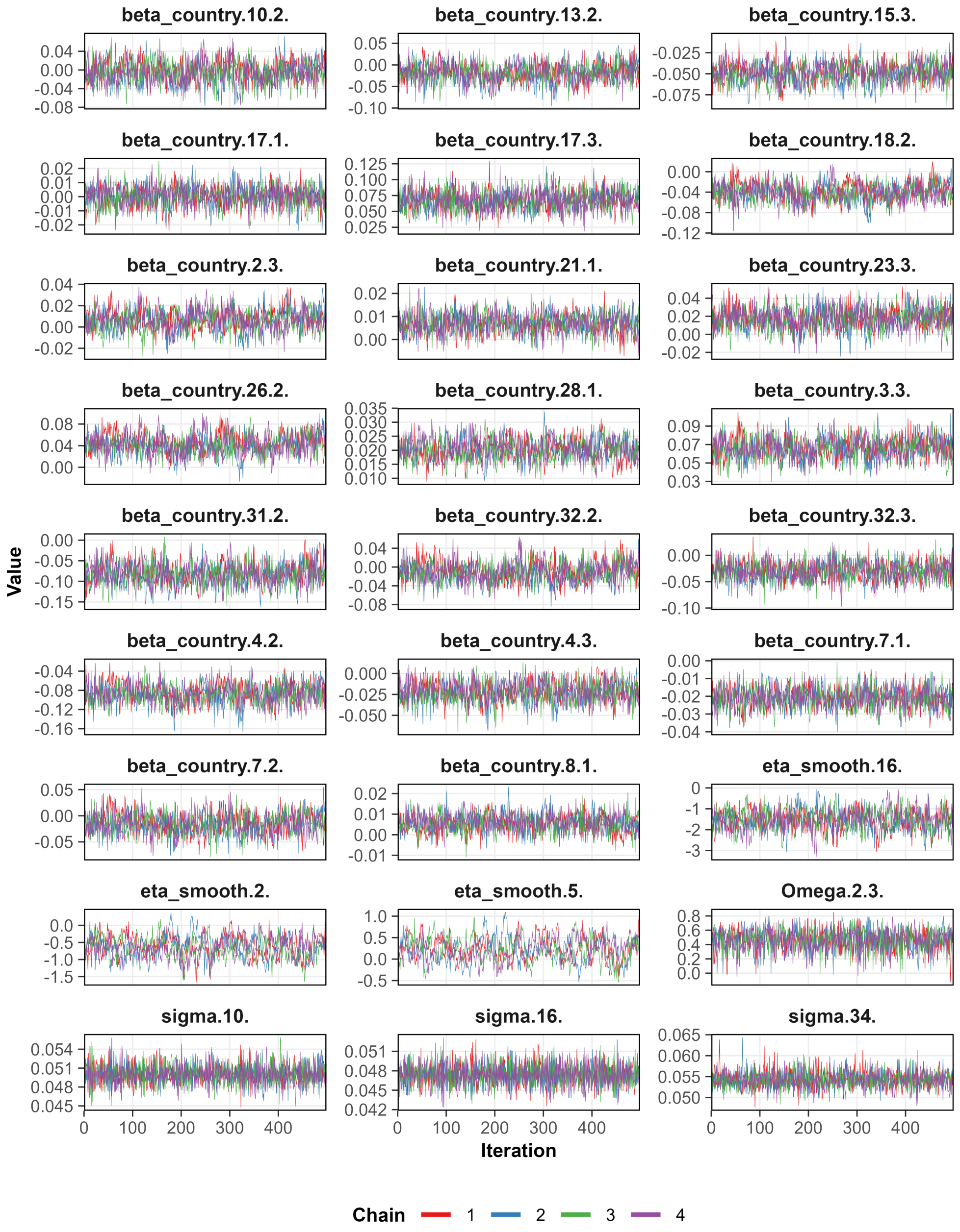

Fig. S5 Traceplots for 27 randomly selected model parameters. Colours indicate different chains.

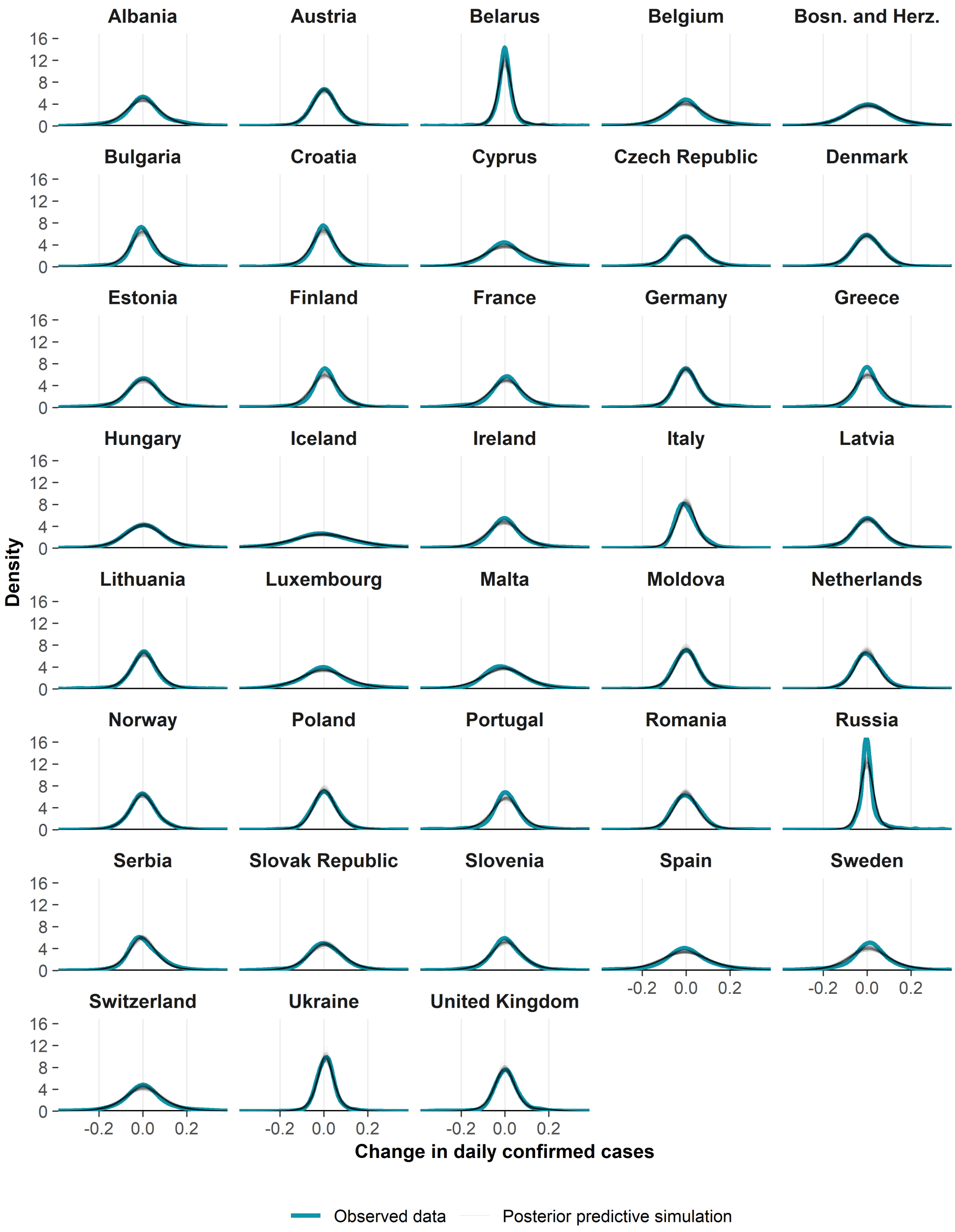

Fig. S6 Country-specific posterior predictive checks for the main model. The solid blue line shows the observed density of changes in daily confirmed cases per country, while the transparent black lines show the posterior predictive simulations per country for the different MCMC draws, thinned to every tenth iteration. The left panel shows results for a model version featuring a normal likelihood and reveals substantial kurtosis.

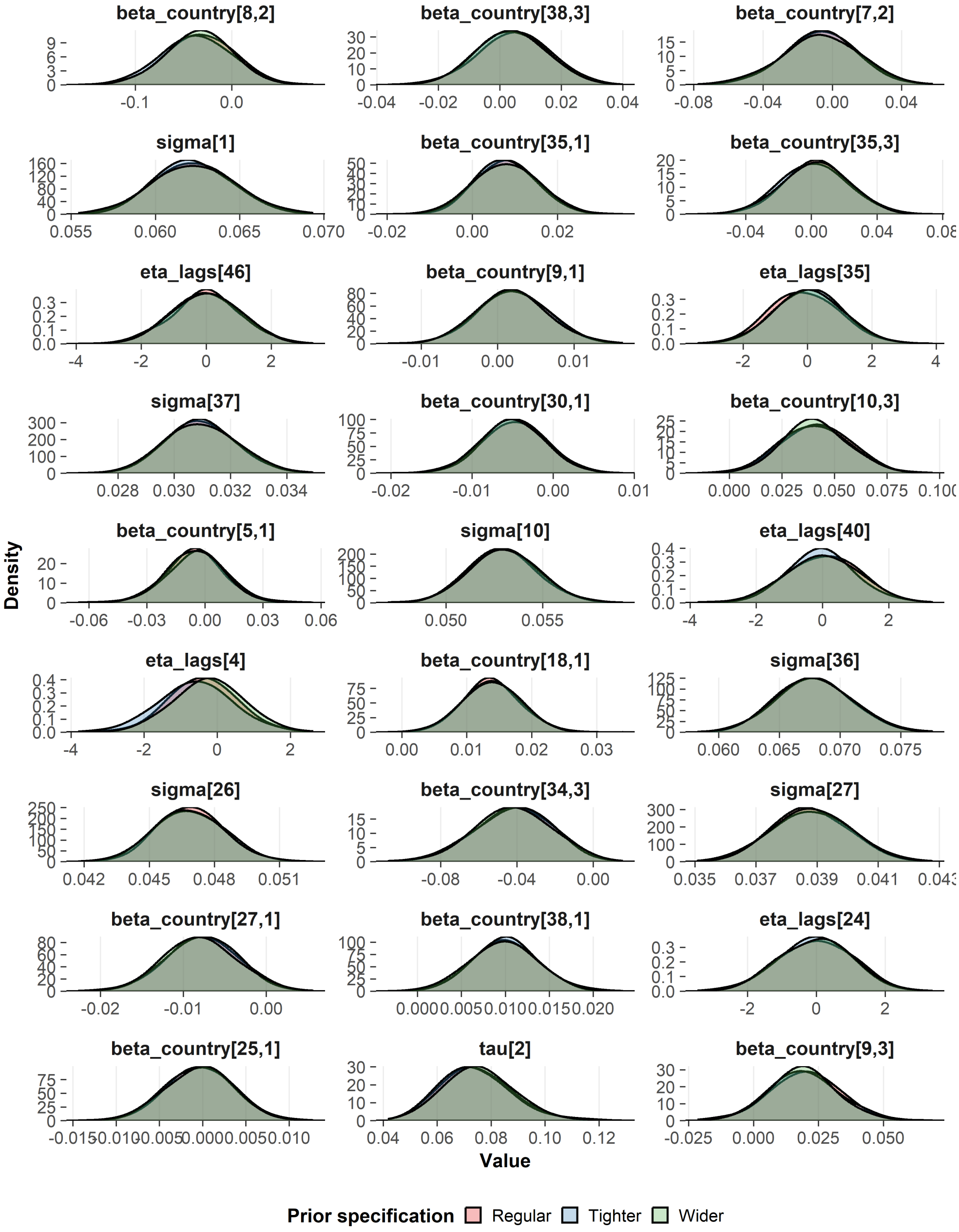

Fig. S7 Prior sensitivity analysis with three different prior specifications, showing that the considered prior only minimally impacts the posterior density of 27 randomly selected model parameters. The red posterior density represents the regular prior specification, as outlined in the main text of this paper. The blue posterior density represents a tighter prior specification, where the scale parameters of relevant priors have been divided by two: $\beta_0 \sim \mathcal{N}(0, 0.5)$, $\boldsymbol{\tau} \sim \mathcal{N}^+(0, 0.1)$, $\alpha \sim \mathcal{N}^+(0, 5)$, $\sigma_c \sim \mathcal{N}^+(0, 0.5)$ and $\lambda \sim \mathcal{N}^+(0, 0.5)$. The green posterior density represents a wider prior specification, where the scale parameters of relevant priors have been multiplied by two: $\beta_0 \sim \mathcal{N}(0, 2)$, $\boldsymbol{\tau} \sim \mathcal{N}^+(0, 0.4)$, $\alpha \sim \mathcal{N}^+(0, 20)$, $\sigma_c \sim \mathcal{N}^+(0, 2)$ and $\lambda \sim \mathcal{N}^+(0, 2)$.

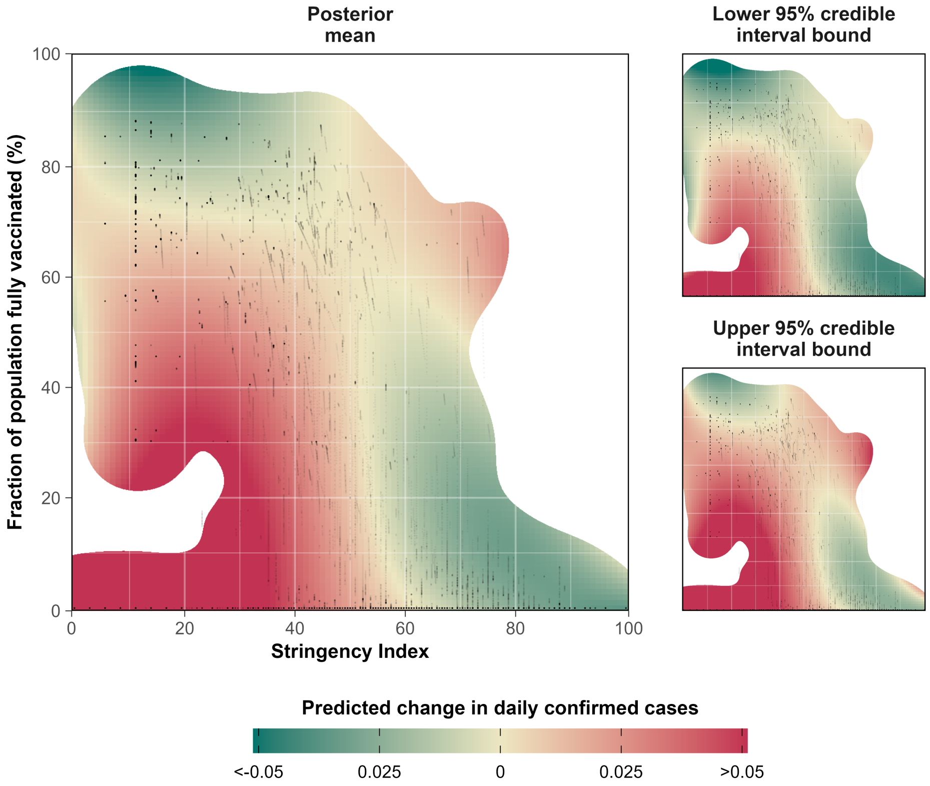

Fig. S8 Alternative version of Fig. 1, upon excluding the country Belarus as a sensitivity analysis.

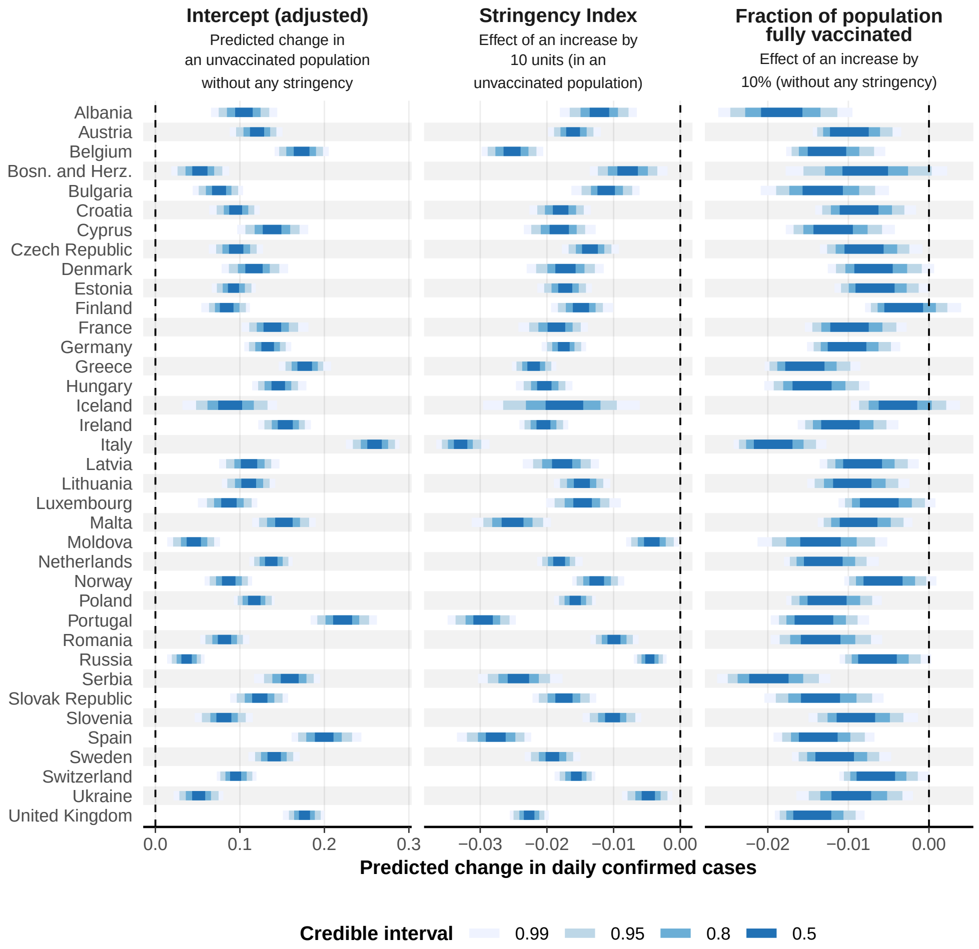

Fig. S9 Alternative version of Fig. 2, upon excluding the country Belarus as a sensitivity analysis.

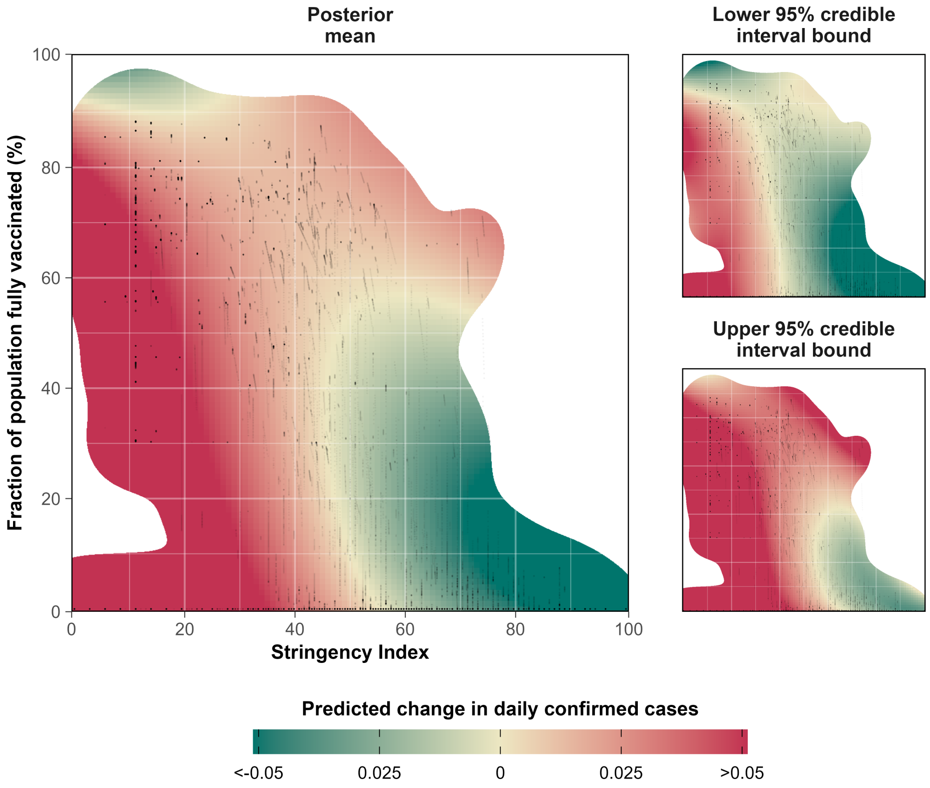

Fig. S10 Alternative version of Fig. 1, upon modelling country-specific temporal variation through a Gaussian process, as a sensitivity analysis. In this alternative analysis, the Gaussian process accounts for any temporal dynamics that cannot be explained by the Stringency Index and the fraction of population fully vaccinated. Such dynamics include gradual changes in reporting, endogenous processes, personal behavioural adjustments and meteorologically-induced seasonality (among others), and might confound the effect of the Stringency Index and the fraction of population fully vaccinated. However, this alternative model specification does not reveal major quantitative differences compared to the original modelling results (Figure 1). The largest differences are situated in the boundary regions of the two-dimensional space, with stronger predicted changes in daily confirmed cases in the alternative analysis. Likely, accounting for temporal dynamics mainly affects these regions as they are data-poor and pertain to a limited amount of countries and periods and, hence, are more sensitive.

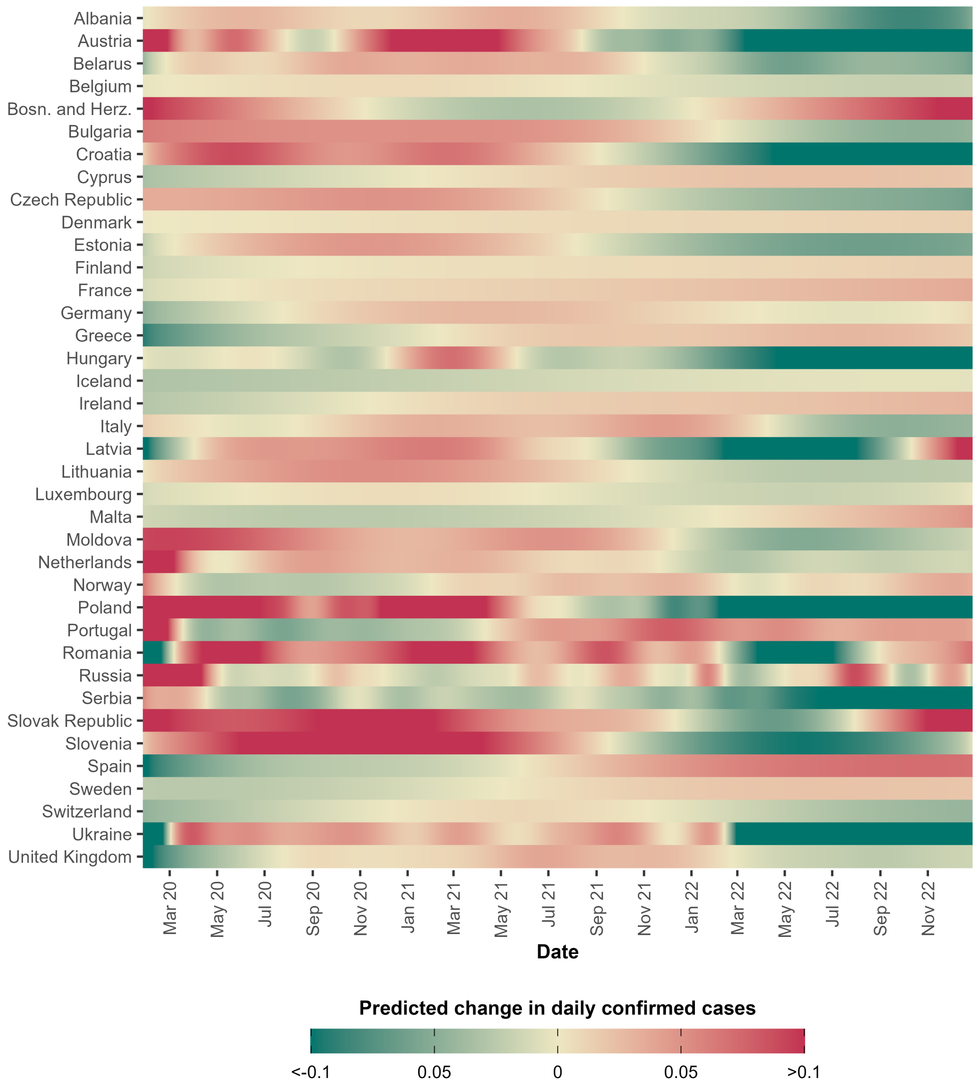

Fig. S11 Posterior mean country-specific temporal variation across the study period, as obtained from the alternative model specification that includes a temporal Gaussian process, as a sensitivity analysis. Green zones correspond to decreasing daily confirmed cases, while red zones correspond to increasing daily confirmed cases. Yellow zones are expected to feature stable numbers of confirmed cases. Note that these are conditional patterns for the temporal Gaussian process only, without the estimated influence of the Stringency Index and the fraction of population fully vaccinated.
